# Extraction of Heart Rate Variability from Smartphone Photoplethysmograms

**DOI:** 10.1155/2015/516826

**Published:** 2015-01-12

**Authors:** Rong-Chao Peng, Xiao-Lin Zhou, Wan-Hua Lin, Yuan-Ting Zhang

**Affiliations:** ^1^Shenzhen Institutes of Advanced Technology, Chinese Academy of Sciences, 1068 Xueyuan Road, Xili Nanshan, Shenzhen, Guangdong 518055, China; ^2^Shenzhen College of Advanced Technology, University of Chinese Academy of Sciences, 1068 Xueyuan Road, Xili Nanshan, Shenzhen, Guangdong 518055, China; ^3^Key Laboratory for Health Informatics of the Chinese Academy of Sciences (HICAS), 1068 Xueyuan Road, Xili Nanshan, Shenzhen, Guangdong 518055, China; ^4^Department of Electronic Engineering, The Chinese University of Hong Kong, Hong Kong

## Abstract

Heart rate variability (HRV) is a useful clinical tool for autonomic function assessment and cardiovascular diseases diagnosis. It is traditionally calculated from a dedicated medical electrocardiograph (ECG). In this paper, we demonstrate that HRV can also be extracted from photoplethysmograms (PPG) obtained by the camera of a smartphone. Sixteen HRV parameters, including time-domain, frequency-domain, and nonlinear parameters, were calculated from PPG captured by a smartphone for 30 healthy subjects and were compared with those derived from ECG. The statistical results showed that 14 parameters (AVNN, SDNN, CV, RMSSD, SDSD, TP, VLF, LF, HF, LF/HF, nLF, nHF, SD1, and SD2) from PPG were highly correlated (*r* > 0.7, *P* < 0.001) with those from ECG, and 7 parameters (AVNN, TP, VLF, LF, HF, nLF, and nHF) from PPG were in good agreement with those from ECG within the acceptable limits. In addition, five different algorithms to detect the characteristic points of PPG wave were also investigated: peak point (PP), valley point (VP), maximum first derivative (M1D), maximum second derivative (M2D), and tangent intersection (TI). The results showed that M2D and TI algorithms had the best performance. These results suggest that the smartphone might be used for HRV measurement.

## 1. Introduction

The heart rate (HR) of human is not constant but varies from one heartbeat to the next. Heart rate variability (HRV) is the physiological phenomenon of tiny fluctuations in the time intervals between heartbeats. It reflects the tenseness and the balance of the sympathetic and the vagus nerve activities and their effects on cardiovascular motion [1]. It is a noninvasive method for assessing the autonomic functions [1]. Numerous publications have validated that abnormal changes of HRV are related to several cardiological and noncardiological diseases like myocardial infarction, diabetic neuropathy, myocardial dysfunction, and tetraplegia [2]. Therefore, HRV is of significant importance and is widely used in clinical application.

HRV is traditionally determined by digital processing of electrocardiograms (ECG). The R-wave peaks of QRS complexes in ECGs are detected by computer algorithms and R-to-R intervals (RRI) are calculated. Then, HRV parameters are computed using time-domain, frequency-domain, and nonlinear methods [2]. However, the traditional measurement of ECG has several limitations: (1) ECG instruments generally require three electrodes attached to specific anatomical positions, which limit the subjects' activities and make them uncomfortable; (2) the electrodes may cause skin irritations for some special subjects with allergies; (3) the ECG instruments are usually operated by specially trained nurses in the hospital and are not suitable for daily use at home. Therefore, new technologies have been developed to measure HRV without ECG, such as photoplethysmography (PPG) [3–5], Finapres (continuous blood pressure monitoring) [6, 7], impedance plethysmography [8, 9], ballistocardiography [10], optical vibrocardiography [11], a microwave sensor [12], or a webcam [13, 14]. In this paper, we demonstrate that HRV can also be extracted from a smartphone.

Mobile phones have already shown promising applications in healthcare service [15]. As the new generation smartphones are becoming more powerful and more popular and with more built-in sensors, the smartphone-based healthcare applications are being rapidly developed [16]. In recent years, a new method was proposed to acquire PPG signals from the built-in camera of a smartphone, in which one only needs to press a finger on the camera lens and capture a video record with the built-in LED flash on [17]. This method is based on the principle that the intensity changes of video frames are associated with the variations of light absorption of blood. When the heart systoles and the capillaries in the fingertip are full of blood, more light is absorbed and the frame becomes darker; likewise, when the heart diastoles and the capillaries in the fingertip are full of less blood, less light is absorbed and the frame becomes brighter. PPG signals can thus be obtained by calculating the intensity changes of the frames, and then several physiological parameters such as HR [17–21], respiratory rate [19], pulse volume [21], and oxygen saturation [19] can be extracted from the PPG signals. With the addition of a microphone to detect the phonocardiogram signal, blood pressure can also be estimated [22]. This method is simple, low-cost, and easy-to-use, with great potential to be used in the healthcare service in the future.

However, to our best knowledge, the extraction of HRV from the smartphone PPG signals has not been well investigated, especially compared with ECG—the golden standard. Therefore, we comprehensively studied the extraction of HRV from smartphone PPG signals and compared the results with an ECG in order to assess the accuracy. Specifically, we used five algorithms to detect the characteristic points of the smartphone PPG signals: peak point (PP), valley point (VP), maximum first derivative (M1D), maximum second derivative (M2D), and tangent intersection (TI). The performances of these algorithms were also compared.

## 2. Methods

### 2.1. Data Acquisition

The experiment was approved by the Institutional Review Board of Shenzhen Institutes of Advanced Technology (registration number: SIAT-IRB-140215-H0040). Thirty subjects participated in the experiment (20 males and 10 females, age: 20–32 years, height: 150–183 cm, and weight: 40–90 kg). All the subjects were healthy and provided their informed consent. They were asked to refrain from caffeine, alcohol, cigarette, or strenuous exercise for 2 hours prior to the study.

In the experiment, all the subjects were instructed to lie in the supine position on a mattress and place their right index finger on the camera lens of an HTC S510e smart phone with the built-in LED flash turned on. A camera application (APP) in the smart phone was used to record the video of the fingertip with a resolution of 320 × 240 pixels at an unfixed sampling rate of 20–30 frames per second (fps). The sampling rate is unfixed due to the CPU processing load. ECG electrodes in the standard configuration were attached to the subjects to measure the ECG signals with a Finometer MIDI (Model II, Finapres Medical Systems B.V., The Netherlands). The ECG signals were digitalized at 200 Hz and automatically stored in the computer by BeatScope Easy software (Finapres Medical Systems B.V., The Netherlands). The experiment lasted at least 5 minutes for each subject and the subject was asked to keep still during this period.

### 2.2. Smartphone PPG Processing

All the data were processed offline. The 3GP format videos recorded by the HTC S510e smart phone were converted into AVI format using Pazera Free 3GP to AVI Converter 1.3 (). All further analysis was performed on the AVI videos in MatLab 7.0 (The Mathworks Inc., USA).

First, an 80 × 80 pixel region in the center of the video image was selected as the region of interesting (ROI). Then, the average intensity of the red channel in the ROI for each individual frame was calculated to generate a time-series waveform (the raw PPG signal). The red channel was chosen because the intensity values of the green and blue channels were often tending to zero and contained no valuable information in most situations. As the smartphone PPG worked in the reflection mode, the generated waveform should be inverted to “normal” mode for further processing [23].

The raw PPG signals were often corrupted by random noise, baseline drifting, and baseline abrupt changes (increase/decrease). Baseline abrupt changes were possibly caused by sudden moves of the finger or sudden changes of the light illumination, or by other unknown reasons. They could not be completely removed by general digital filters as they contained wide-band frequency components. We used a statistics method to solve this problem. First, we calculated the difference of the raw signal and then removed the outliers out of the range mean ± 5 × standard deviation (SD) and interpolated new values using cubic spline interpolation. At last, we reconstructed the new PPG signal by summation, the inverse of the difference. The range mentioned above was determined empirically, which meant that the probability of the outliers was 5.7330 × 10^−7^ if the difference of the PPG was normally distributed. It was the best range according to our data and could be adjusted if required.

The random noise and baseline drifting were reduced by a zero-phase Butterworth low-pass filter with cutoff frequency of 10 Hz and a zero-phase Butterworth high-pass filter with cutoff frequency of 0.5 Hz, respectively. Zero phase filters were implemented by filtering the signal both forward and backward to eliminate phase distortion.

The PPG signals were then resampled to 800 Hz with cubic spline interpolation to increase the temporal resolution. For each cardiac circle, five algorithms were used to obtain the pulse-to-pulse interval (PPI) by detection of five different characteristic points, as illustrated in Figure 2.

*(i) Maximum First Derivative (M1D).* It is the location of the maximum value of the first derivative of the PPG signal [24]. The derivative is calculated by a five-point central difference equation (1).

*(ii) Peak Point (PP).* It is the location of the maximum amplitude in the PPG signal following the M1D point [24].

*(iii) Valley Point (VP).* It is the location of the minimum amplitude in the PPG signal preceding the M1D point [24].

*(iv) Maximum Second Derivative (M2D).* It is the location of the maximum value of the second derivative of the PPG signal [25]. The second derivative is calculated using a five-point central difference equation (1) and a subsequent seven-point central difference equation (2). Consider

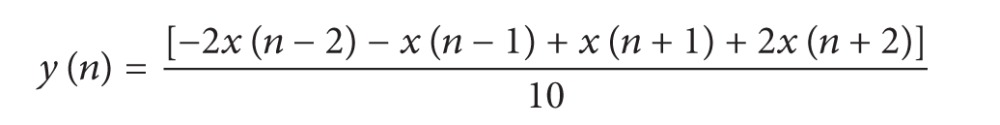
(1)

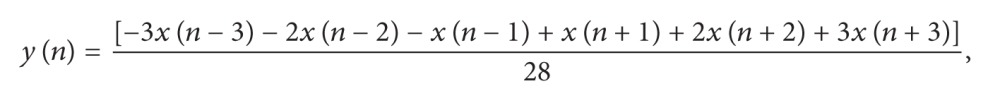
(2)
where *x*(*n*) is the input signal and *y*(*n*) is the output signal.

*(v) Tangent Intersection (TI).* It is the location of the intersection of the tangent line at the M1D point and the horizontal line passing the valley point [25]. The tangent line is the fitted line of five points centered at the M1D point.

### 2.3. ECG Processing

The ECG signals were first passed through a finite impulse response (FIR) low-pass filter with cutoff frequency of 11 Hz and then a FIR high-pass filter with cutoff frequency of 5 Hz to reduce most of the noise and interference [26]. Thereafter, they were resampled to 800 Hz with cubic spline interpolation to increase the temporal resolution. R-wave peak detection was performed using Pan and Tompkins' algorithm [26] and RRIs were obtained as the difference of successive R-wave peak locations. For both PPG and ECG signals, missed beats and false beats were manually identified and adjusted. An example of the obtained RRI and PPIs is shown in Figure 3.

### 2.4. HRV Parameters Calculation

Table 1 lists some commonly used HRV parameters. These parameters often refer to a professional term, NN intervals (“normal-to-normal intervals”), which means that only regular heartbeats should be considered [2]. Therefore, ectopic beats of the RRI/PPI series were removed and replaced by cubic spline interpolation before HRV parameters calculation.

*(i) Time-Domain Parameters.* Seven parameters were calculated from RRI and PPI series in time domain: AVNN, CV, SDNN, RMSSD, SDSD, NN50, and pNN50, as described in Table 1.

*(ii) Frequency-Domain Parameters.* The RRI and PPI series were evenly resampled at 4 Hz using cubic spline interpolation, and the DC component was removed by subtracting the mean of the series. Then, a 16th order autoregressive (AR) model was employed to estimate the power spectral density. Seven parameters were calculated: TP, VLF, LF, HF, LF/HF, nLF, and nHF, as described in Table 1. The ranges of different frequency bands were in accordance with the standard definition [2].

*(iii) Poincaré Parameters.* The Poincaré plot is one of the most widely used methods for nonlinear HRV analysis. It is a plot of each RRI/PPI against its previous one. Two parameters were calculated from the Poincaré plot, SD1 and SD2, as described in Table 1.

### 2.5. Statistics Analysis

HRV parameters derived from smartphone PPG were compared with the corresponding parameters derived from ECG. The Pearson correlation coefficients were calculated and the linear regression equations were obtained. A *P* value < 0.05 was considered statistically significant.

The agreement between the two devices (smartphone and ECG) was assessed using Bland-Altman method [27]. The limit of agreement (LOA) was defined as bias ± 1.96 × SD (3)–(5) [27] and a Bland-Altman ratio (BAR) was defined as the ratio of half the range of limits of agreement to the mean of the pairwise measurement means (6). Agreements were ranked as good (BAR < 10%), moderate (10% ≤ BAR < 20%), or insufficient (BAR ≥ 20%) [28]. Acceptable limit (AL) of agreement was defined as 20% of the mean of the pairwise measurement means (7), as there are wide interindividual variations for HRV measurement [2] and a limit greater than 20% is generally considered unacceptable. Consider

(3) $$\begin{array}{c} \text{LOA}=\text{Bias}\pm 1.96\text{SD} \end{array}$$

(4) $$\begin{array}{c} \text{Bias}=\frac{1}{n}\overset{n}{\underset{i=1}{\sum }}\left(y_{i}-x_{i}\right) \end{array}$$

(5) $$\begin{array}{c} \text{SD}=\sqrt{\frac{1}{n-1}\overset{n}{\underset{i=1}{\sum }}{\left(y_{i}-x_{i}-\text{Bias}\right)}^{2}} \end{array}$$

(6) $$\begin{array}{c} \text{BAR}=\frac{1.96\text{SD}}{\left(1/n\right)\sum_{i=1}^{n}\left(1/2\right)\left(x_{i}+y_{i}\right)} \end{array}$$

(7) $$\begin{array}{c} \mathrm{AL}=\pm \frac{1}{n}\overset{n}{\underset{i=1}{\sum }}\frac{1}{2}\left(x_{i}+y_{i}\right)\times 20\%, \end{array}$$

where *x* and *y* are the HRV parameters derived from the ECG and the smartphone, respectively, and *n* is the number of subjects.

## 3. Results

### 3.1. Agreement Analysis

Table 2 shows the Pearson correlation coefficients and linear regression equations between HRV parameters derived from the smartphone and the ECG. It was found that the correlation coefficients were >0.6 for all parameters except NN50 and pNN50. For time-domain parameters, the correlation coefficients of AVNN, CV, and SDNN were higher than those of RMSSD and SDSD. For frequency-domain parameters, all parameters showed strong correlations (*r* > 0.9, *P* < 0.001). For nonlinear parameters, SD2 exhibited higher correlation (*r* > 0.9, *P* < 0.001) than SD1 (*r* > 0.5, *P* > 0.001). Nevertheless, a good correlation does not mean a good agreement as either adding a constant or multiplying a factor will still yield a good correlation. A good method for assessing agreement is Bland-Altman analysis.

**Figure 1:**
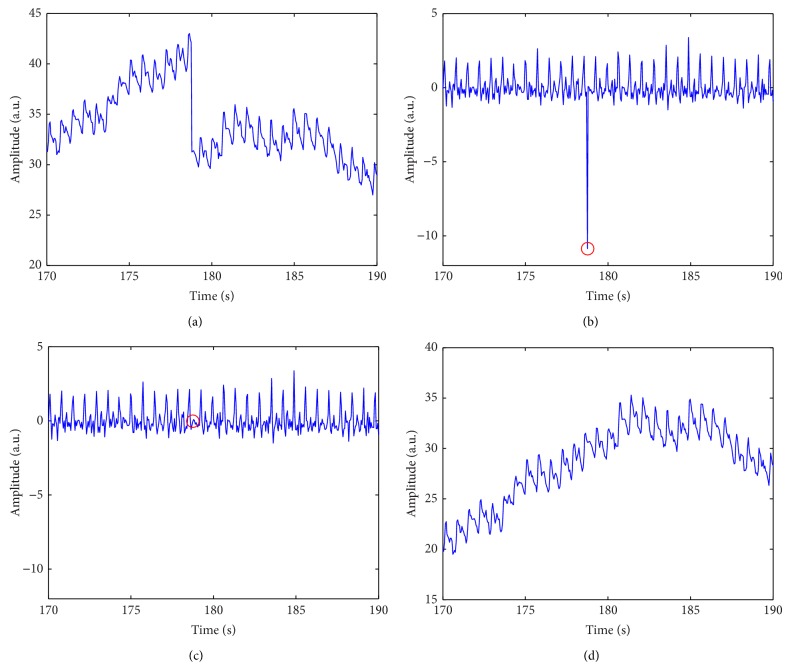
An example of outlier removal. (a) A raw smartphone photoplethysmogram with abrupt change. (b) The difference of the signal in panel (a). The circle shows the location of the outlier. (c) The outlier was removed and replaced with a new value using cubic spline interpolation. (d) The new smartphone photoplethysmogram without abrupt change.

**Figure 2:**
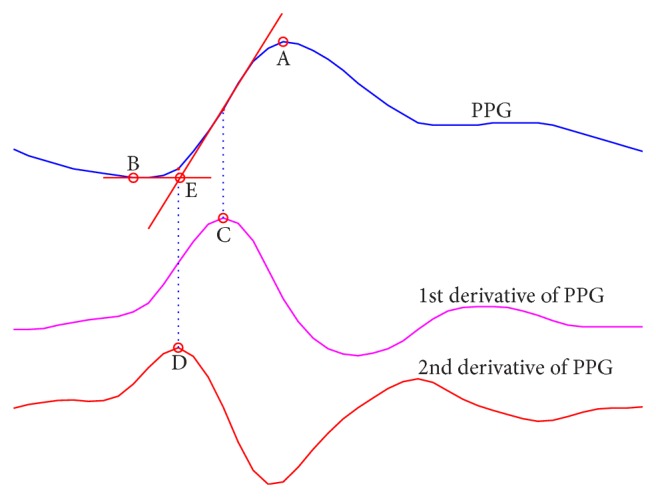
Illustration of five characteristic points including A, the peak point; B, the valley point; C, the maximum first derivative; D, the maximum second derivative; and E, the tangent intersection.

**Figure 3:**
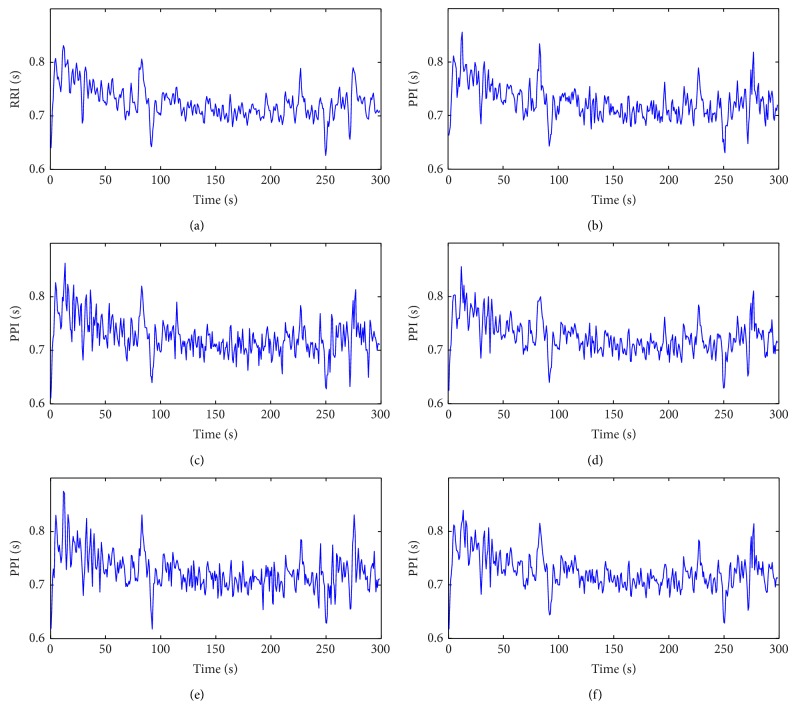
Comparison of HRV derived from the smartphone and the electrocardiograph for one subject. (a) R-to-R intervals (RRI) derived from the electrocardiogram. (b)–(f) Pulse-to-pulse intervals (PPI) derived from the smartphone photoplethysmogram, using the characteristic points determined by (b) peak point, (c) valley point, (d) maximum first derivative, (e) maximum second derivative, and (f) tangent intersection.

Table 3 shows the Bland-Altman analysis of HRV parameters derived from the smartphone and the ECG. For the sake of simplicity, we speak of good/moderate agreement if three of the five algorithms were in good/moderate agreement. It was found that all the time-domain parameters showed insufficient agreements (BAR ≥ 20%), but the AVNN showed excellent agreement (BAR < 1%), indicating that smartphone-derived HR can be a surrogate of ECG-derived HR. This result was in line with Gregoski et al.'s [20] and Matsumura and Yamakoshi's [21]. It was also found that all the frequency-domain parameters were in moderate agreement (BAR < 20%) except for TP and nLF which were in good agreement (BAR < 10%). TP, VLF, HF, and nHF were overestimated (bias > 0), while LF, LF/HF, and nLF were underestimated (bias < 0), implying that the smartphone-derived HRV contains more noise, which can be observed in detail in Figure 1. For nonlinear parameters, SD2 showed good agreement (BAR < 10%) and SD1 showed insufficient agreement (BAR ≥ 20%).

**Figure 4:**
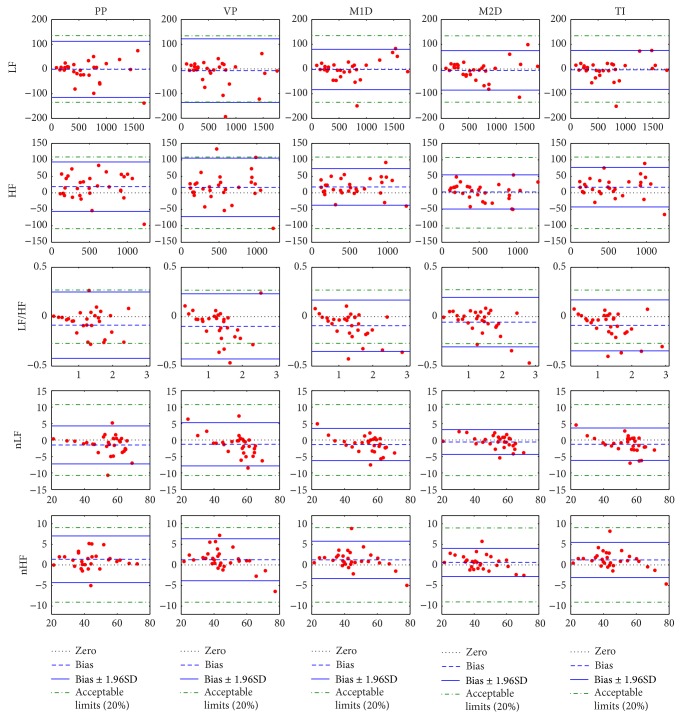
Bland-Altman plots of HRV parameters derived from the smartphone and the electrocardiograph. For each plot, the horizontal axis represents the mean of HRV parameters derived from smartphone and electrocardiograph, while the vertical axis represents the difference between HRV parameters derived from smartphone and electrocardiograph. The five columns correspond to five different algorithms: PP, peak point; VP, valley point; M1D, maximum first derivative; M2D, maximum second derivative; and TI, tangent intersection. LF, low frequency power; HF, high frequency power; LF/HF, ratio of LF to HF; nLF, normalized LF = LF/(TP − VLF); and nHF, normalized HF = HF/(TP − VLF).

As shown in Table 3, a total number of 7 parameters (AVNN, TP, VLF, LF, HF, nLF, and nHF) were within the acceptable limits.  Figure 4 shows the Bland-Altman plots of different frequency components of HRV which are commonly used for assessing the autonomic functions. It was found that the limits of agreement for LF, HF, nLF, and nHF were all within their corresponding acceptable limits, meaning that the discrepancies between the smartphone PPG and the ECG for LF, HF, nLF, and nHF were not considerable. It was also found that the lower limit of agreement for LF/HF is out of the range of acceptable limits.

### 3.2. Algorithms Comparison

In terms of both bias and SD, we analyzed these data satisfying the condition BAR < 20% in Table 3 to evaluate the performance of the five algorithms mentioned above. For each HRV parameter, the best two algorithms with the least bias or SD would gain a star “^*^” each. The overall performance was graded according to the number of total stars. As shown in Table 4, the M2D and the TI algorithms were better than the others, the PP and the VP were the worst, and the M1D was in the middle.

## 4. Discussion

As previously mentioned, no researches have been reported to measure HRV with smartphone PPG. Nevertheless, many researches have been reported to measure HRV with traditional PPG (tPPG, i.e., a pulse oximeter), which can provide valuable information for our work. A good review of tPPG-derived HRV can be found in [28], where the authors commented that it was controversial whether the tPPG-derived HRV was a surrogate of the ECG-derived HRV. Although a number of publications reported universally good agreement between tPPG-derived HRV and ECG-derived HRV for all parameters, many other studies found that short-term parameters were more susceptible to disagreement between tPPG and ECG than long-term parameters. Our results are in accordance with the latter. As shown in Table 2, the coefficients of long-term parameters (AVNN, SDNN, and CV) are higher than the short-term parameters (RMSSD, SDSD, NN50, and pNN50). As shown in Table 3, the low-frequency parameters are in higher agreement than the high-frequency parameters, as the bias of LF is less than that of the HF and their BARs have no significant difference. The nonlinear parameter SD2 presenting the level of long-term HRV is in good agreement between ECG and smartphone PPG while SD1 presenting the level of short-term HRV is in insufficient agreement.

The detection of the characteristic points is also an impact factor for the accuracy of smartphone-derived HRV measurement [4]. As the morphology of pulse wave changes over time, an algorithm less sensitive to morphology variation will produce better accuracy. We graded these five algorithms: PP, VP, M1D, M2D, and TI. Our results indicate that M2D and TI algorithms are better than the others, which are in accordance with Chiu et al.'s [25], but they are slightly different from Posada-Quintero et al.'s [29]. Posada-Quintero et al. stated that the TI algorithm was better than the VP and M2D algorithms. This slight difference is possibly due to different algorithm details and different evaluation criteria. Another interesting research found that PP was more sensitive to waveform distortion than VP and M1D when the peripheral pulse was affected by cold temperature [24]. Overall, M2D and TI algorithms are better than PP, VP, and M1D. The fact that many researchers used the PP algorithm is worthy of attention [3, 4, 30, 31]. In addition, none of the algorithms is perfect or error-free. A manual correction is usually required by visual inspection on the computer screen, which is time-consuming. More efficiency algorithms are needed.

A possible consideration of the smartphone-based HRV analysis is the sampling rate. Our HTC smartphone has a sampling rate of approximately 20–30 Hz that may be considered not suitable for HRV analysis. In fact, the spectrum of the pulse signals has the vast majority power in the range of 0~10 Hz [32]. A sampling rate of 20 Hz is not less than the Nyquist rate and the temporal resolution can be improved by interpolation, which was confirmed by Sun et al.'s experiments in [14] where they compared different sampling rates of 200, 100, 50, and 20 fps and their results revealed no significant differences of these sampling rates. Moreover, as the new generation smartphones have more powerful CPUs and higher speed cameras, the sampling rate will be improved for better performance.

The color channel should be also considered. In the processing of the recorded video, most previous studies calculated the intensity of the green channel in RGB color model [17–19], because (oxy)haemoglobin absorbs more green light than red light and penetrates sufficiently deep into the skin as compared to blue light [33]. We chose the red channel over the green or blue channel, because we observed that the pixel values in green and blue channels were tending to zero and the changes of the red channel are more pronounced than the green and blue channels in most situations. Chandrasekaran et al. also observed that the prominent color was red and they used the red channel in their research [22]. Grimaldi et al. demonstrated that the distribution of the pixels in the green channel was not uniform for different models of the smartphones and the only channel that had similar characteristics was the red one [34]. This suggests that heterogeneous characteristics of different cameras in different smartphones should be taken into account and more robust algorithms are required. Some potential alternatives are principal component analysis (PCA), independent component analysis (ICA), or using other color models (e.g., CMY color model, HSI color model, and YUV color model).

Motion artifacts are another complicated problem and are tough to deal with. To our best knowledge, none of the reported studies have solved this problem very well. In our experiments, the subjects were instructed to lie on a mattress and keep their fingers as still as possible to minimize the motion artifacts. This is not practical in daily life as short-time HRV testing usually takes 5 minutes that seems so long time for keeping still. Therefore, efficient motion-resistant algorithms are required. Several motion artifacts detection algorithms in pulse oximeters could be applied in smartphone-based HRV analysis [35, 36].

## 5. Conclusion

Traditional ECG recordings require electrodes attached to body surface and are operated by specially trained nurses in the hospital. The new smartphone-based technology requires no more than placing a finger on the camera lens of a smartphone. It is low-cost and easy-to-use and can be used in daily life out of hospital. In the present study, we quantitatively investigated the measurement of HRV based on smartphone technology and compared the results with those derived from a standard ECG to assess the accuracy. The results suggest that the smartphone can be of potential use for HRV measurement at resting and would be applied in low-cost healthcare applications.

## Figures and Tables

**Table 1:** Commonly used HRV parameters.

Parameter	Description
*Time-domain *	
AVNN	Average of all NN intervals
CV	Coefficient of variation of NN intervals. The ratio of the standard deviation to the mean.
SDNN	Standard deviation of all NN intervals.
SDANN	Standard deviation of the averages of NN intervals in all 5-minute segments of the entire recording.
RMSSD	Root mean square of successive differences between adjacent NN intervals.
SDSD	Standard deviation of successive differences between adjacent NN intervals.
NN50	Number of pairs of successive NN intervals that differ by more than 50 minutes.
pNN50	Proportion of NN50 divided by total number of NN intervals.
*Frequency-domain (5 minutes) *	
TP	Total power (≤0.4 Hz)
VLF	Very low frequency power (≤0.04 Hz)
LF	Low frequency power (0.04–0.15 Hz)
HF	High frequency power (0.15–0.4 Hz)
LF/HF	Ratio of LF to HF
nLF	Normalized LF = LF/(TP − VLF)
nHF	Normalized HF = HF/(TP − VLF)
*Nonlinear Analysis *	
SD1	Standard deviation of short diagonal axis in Poincaré plot
SD2	Standard deviation of long diagonal axis in Poincaré plot

**Table 2:** Pearson's correlation coefficients and linear regression equations between HRV parameters derived from the smartphone and the electrocardiograph.

Parameter	PP	VP	M1D	M2D	TI
AVNN (ms)	1.000 (*P* < 0.001) *y* = 1.000*x* + 0.072	1.000 (*P* < 0.001) *y* = 1.000*x* + 0.019	1.000 (*P* < 0.001) *y* = 1.000*x* − 0.084	1.000 (*P* < 0.001) *y* = 1.000*x* − 0.367	1.000 (*P* < 0.001) *y* = 1.000*x* − 0.097

SDNN (ms)	0.722 (*P* = 0.000) *y* = 0.912*x* + 33.708	0.902 (*P* = 0.000) *y* = 0.865*x* + 26.556	0.933 (*P* = 0.000) *y* = 0.891*x* + 19.641	0.859 (*P* = 0.000) *y* = 0.785*x* + 35.635	0.916 (*P* = 0.000) *y* = 0.875*x* + 20.304

CV (%)	0.703 (*P* < 0.001) *y* = 0.756*x* + 4.519	0.881 (*P* < 0.001) *y* = 0.893*x* + 2.675	0.920 (*P* < 0.001) *y* = 0.918*x* + 1.934	0.826 (*P* < 0.001) *y* = 0.827*x* + 3.561	0.900 (*P* < 0.001) *y* = 0.903*x* + 2.008

RMSSD (ms)	0.596 (*P* = 0.001) *y* = 1.034*x* + 66.019	0.713 (*P* < 0.001) *y* = 0.801*x* + 57.427	0.780 (*P* < 0.001) *y* = 0.811*x* + 44.095	0.629 (*P* < 0.001) *y* = 0.677*x* + 74.746	0.731 (*P* < 0.001) *y* = 0.796*x* + 43.870

SDSD (ms)	0.596 (*P* = 0.001) *y* = 1.035*x* + 66.086	0.713 (*P* < 0.001) *y* = 0.801*x* + 57.497	0.780 (*P* < 0.001) *y* = 0.811*x* + 44.152	0.630 (*P* < 0.001) *y* = 0.677*x* + 74.839	0.732 (*P* < 0.001) *y* = 0.796*x* + 43.924

NN50	0.254 (*P* = 0.176) *y* = 0.203*x* + 90.688	0.285 (*P* = 0.127) *y* = 0.184*x* + 79.417	0.292 (*P* = 0.118) *y* = 0.215*x* + 73.810	0.081 (*P* = 0.669) *y* = 0.060*x* + 96.888	0.391 (*P* = 0.033) *y* = 0.306*x* + 66.480

pNN50 (%)	0.415 (*P* = 0.022) *y* = 0.313*x* + 27.188	0.508 (*P* = 0.004) *y* = 0.287*x* + 23.540	0.513 (*P* = 0.004) *y* = 0.309*x* + 21.844	0.513 (*P* = 0.004) *y* = 0.309*x* + 21.844	0.513 (*P* = 0.004) *y* = 0.309*x* + 21.844

TP (ms2)	1.000 (*P* < 0.001) *y* = 1.002*x* + 20.325	0.999 (*P* < 0.001) *y* = 1.001*x* + 12.614	1.000 (*P* < 0.001) *y* = 1.009*x* + 6.481	1.000 (*P* < 0.001) *y* = 0.998*x* + 4.521	1.000 (*P* < 0.001) *y* = 1.006*x* + 6.888

VLF (ms2)	0.996 (*P* < 0.001) *y* = 1.002*x* + 5.276	0.995 (*P* < 0.001) *y* = 0.996*x* + 9.597	0.998 (*P* < 0.001) *y* = 0.999*x* + 4.785	0.998 (*P* < 0.001) *y* = 0.992*x* + 7.631	0.998 (*P* < 0.001) *y* = 1.003*x* + 2.669

LF (ms2)	0.992 (*P* < 0.001) *y* = 1.011*x* − 8.803	0.989 (*P* < 0.001) *y* = 0.993*x* − 2.426	0.996 (*P* < 0.001) *y* = 1.022*x* − 16.348	0.996 (*P* < 0.001) *y* = 0.997*x* − 3.807	0.996 (*P* < 0.001) *y* = 1.009*x* − 9.753

HF (ms2)	0.993 (*P* < 0.001) *y* = 0.982*x* + 28.252	0.990 (*P* < 0.001) *y* = 0.986*x* + 24.236	0.996 (*P* < 0.001) *y* = 1.002*x* + 17.071	0.997 (*P* < 0.001) *y* = 0.981*x* + 12.937	0.996 (*P* < 0.001) *y* = 0.997*x* + 18.848

LF/HF	0.963 (*P* < 0.001) *y* = 0.883*x* + 0.078	0.967 (*P* < 0.001) *y* = 0.850*x* + 0.111	0.982 (*P* < 0.001) *y* = 0.871*x* + 0.088	0.984 (*P* < 0.001) *y* = 0.874*x* + 0.120	0.981 (*P* < 0.001) *y* = 0.882*x* + 0.075

nLF (%)	0.968 (*P* < 0.001) *y* = 0.919*x* + 2.896	0.967 (*P* < 0.001) *y* = 0.812*x* + 8.941	0.982 (*P* < 0.001) *y* = 0.873*x* + 5.539	0.988 (*P* < 0.001) *y* = 0.920*x* + 3.754	0.981 (*P* < 0.001) *y* = 0.872*x* + 5.673

nHF (%)	0.977 (*P* < 0.001) *y* = 0.969*x* + 2.760	0.985 (*P* < 0.001) *y* = 0.889*x* + 6.224	0.986 (*P* < 0.001) *y* = 0.925*x* + 4.565	0.992 (*P* < 0.001) *y* = 0.946*x* + 3.027	0.988 (*P* < 0.001) *y* = 0.926*x* + 4.509

SD1 (ms)	0.596 (*P* = 0.001) *y* = 1.035*x* + 46.728	0.713 (*P* < 0.001) *y* = 0.801*x* + 40.656	0.780 (*P* < 0.001) *y* = 0.811*x* + 31.219	0.630 (*P* < 0.001) *y* = 0.677*x* + 52.918	0.732 (*P* < 0.001) *y* = 0.796*x* + 31.058

SD2 (ms)	0.920 (*P* < 0.001) *y* = 0.922*x* + 20.291	0.986 (*P* < 0.001) *y* = 0.934*x* + 14.018	0.989 (*P* < 0.001) *y* = 0.955*x* + 9.982	0.978 (*P* < 0.001) *y* = 0.898*x* + 18.234	0.988 (*P* < 0.001) *y* = 0.953*x* + 10.075

*x*: HRV parameters derived from an electrocardiograph and *y*: HRV parameters derived from a smartphone using five different algorithms. PP: peak point; VP: valley point; M1D: maximum first derivative; M2D: maximum second derivative, and TI: tangent intersection. A *P* value <0.05 was considered statistically significant. HRV parameters are explained in Table 1.

**Table 3:** Bland-Altman analysis of HRV parameters derived from the smartphone and the electrocardiograph.

Parameter	PP	VP	M1D	M2D	TI
AVNN (ms)	−0.05 ± 0.68^*^ BAR = 0.07%	−0.12 ± 0.54^*^ BAR = 0.06%	−0.06 ± 0.55^*^ BAR = 0.06%	−0.05 ± 0.55^*^ BAR = 0.04%	−0.09 ± 0.51^*^ BAR = 0.05%

SDNN (ms)	28.39 ± 31.26 BAR = 41.85%	18.40 ± 15.48 BAR = 22.21%	13.03 ± 12.85 BAR = 19.17%	22.65 ± 12.85 BAR = 25.51%	12.76 ± 14.37 BAR = 21.49%

CV (%)	2.95 ± 2.96 BAR = 37.53%	1.99 ± 1.81 BAR = 24.47%	1.41 ± 1.48 BAR = 20.79%	2.45 ± 1.48 BAR = 28.48%	1.39 ± 1.66 BAR = 23.32%

RMSSD (ms)	67.84 ± 61.35 BAR = 70.14%	46.77 ± 35.77 BAR = 46.50%	33.96 ± 29.77 BAR = 42.22%	57.44 ± 29.77 BAR = 47.93%	32.95 ± 33.85 BAR = 48.35%

SDSD (ms)	67.95 ± 61.47 BAR = 70.17%	46.84 ± 35.83 BAR = 46.50%	34.01 ± 29.82 BAR = 42.21%	57.53 ± 29.82 BAR = 47.92%	33.00 ± 33.91 BAR = 48.35%

NN50	57.57 ± 57.65 BAR = 81.98%	45.53 ± 53.24 BAR = 82.80%	41.20 ± 54.79 BAR = 88.18%	57.87 ± 54.79 BAR = 88.22%	37.67 ± 52.02 BAR = 86.17%

pNN50 (%)	17.88 ± 17.79 BAR = 79.07%	13.87 ± 15.82 BAR = 77.17%	12.47 ± 15.82 BAR = 79.91%	12.47 ± 15.82 BAR = 79.91%	12.47 ± 15.82 BAR = 79.91%

TP (ms2)	23.82 ± 63.56 BAR = 3.82%	14.55 ± 75.59 BAR = 4.56%	20.67 ± 56.93^*^ BAR = 3.42%	0.52 ± 56.93^*^ BAR = 2.55%	17.10 ± 53.88^*^ BAR = 3.24%

VLF (ms2)	6.30 ± 81.19 BAR = 18.96%	8.08 ± 93.97 BAR = 21.90%	4.25 ± 67.45^*^ BAR = 15.79%	4.36 ± 67.45^*^ BAR = 14.66%	4.10 ± 67.97^*^ BAR = 15.91%

LF (ms2)	−1.22 ± 113.36 BAR = 16.83%	−7.05 ± 128.70 BAR = 19.19%	−1.77 ± 81.75^*^ BAR = 12.14%	−5.67 ± 81.75^*^ BAR = 11.89%	−3.39 ± 78.84^*^ BAR = 11.73%

HF (ms2)	18.69 ± 75.18^*^ BAR = 13.82%	16.59 ± 88.85^*^ BAR = 16.37%	17.98 ± 55.56^*^ BAR = 10.22%	2.71 ± 55.56^*^ BAR = 9.69%	17.12 ± 60.00^*^ BAR = 11.05%

LF/HF	−0.09 ± 0.34 BAR = 24.89%	−0.10 ± 0.33 BAR = 24.55%	−0.09 ± 0.26 BAR = 19.30%	−0.06 ± 0.26 BAR = 18.35%	−0.09 ± 0.26 BAR = 19.13%

nLF (%)	−1.50 ± 5.75^*^ BAR = 10.72%	−1.30 ± 6.56^*^ BAR = 12.19%	−1.36 ± 4.89^*^ BAR = 9.09%	−0.60 ± 4.89^*^ BAR = 6.98%	−1.28 ± 4.93^*^ BAR = 9.16%

nHF (%)	1.37 ± 5.68^*^ BAR = 12.55%	1.25 ± 5.10^*^ BAR = 11.28%	1.23 ± 4.53^*^ BAR = 10.01%	0.62 ± 4.53^*^ BAR = 7.58%	1.20 ± 4.28^*^ BAR = 9.46%

SD1 (ms)	48.05 ± 43.47 BAR = 70.17%	33.12 ± 25.33 BAR = 46.50%	24.05 ± 21.08 BAR = 42.21%	40.68 ± 21.08 BAR = 47.92%	23.33 ± 23.97 BAR = 48.35%

SD2 (ms)	14.35 ± 17.58 BAR = 21.12%	8.98 ± 7.49 BAR = 9.30%	6.54 ± 6.51^*^ BAR = 8.21%	10.50 ± 6.51 BAR = 11.79%	6.47 ± 6.74^*^ BAR = 8.50%

Data are presented as bias ± 1.96 standard deviation (SD). ^*^Bias ± 1.96 SD within the acceptable limits. BAR: Bland-Altman ratio, PP: peak point, VP: valley point, M1D: maximum first derivative, M2D: maximum second derivative, and TI: tangent intersection. HRV parameters are explained in Table 1.

**Table 4:** Comparison of five algorithms for detection of characteristic points.

Parameter	PP	VP	M1D	M2D	TI
AVNN					
Bias	∗	—	—	∗	—
SD	—	—	—	∗	∗
TP					
Bias	—	∗	—	∗	—
SD	—	—	—	∗	∗
VLF					
Bias	—	—	∗	—	∗
SD	—	—	∗	∗	—
LF					
Bias	∗	—	∗	—	—
SD	—	—	—	∗	∗
HF					
Bias	—	∗	—	∗	—
SD	—	—	∗	∗	—
LF/HF					
Bias	—	—	—	∗	∗
SD	—	—	—	∗	∗
nLF					
Bias	—	—	—	∗	∗
SD	—	—	∗	∗	—
nHF					
Bias	—	—	—	∗	∗
SD	—	—	—	∗	∗
SD2					
Bias	—	—	∗	—	∗
SD	—	—	∗	—	∗

Total stars	2	2	7	14	11

PP: peak point, VP: valley point, M1D: maximum first derivative, M2D: maximum second derivative, and TI: tangent intersection. SD: standard deviation. HRV parameters are explained in Table 1.
